# Impaired comprehension of diagnostic procedures and medication instructions in a quick diagnostic unit setting

**DOI:** 10.1186/1757-7241-21-S2-A30

**Published:** 2013-09-09

**Authors:** Lisa Nebelin Hvidt, Kim Madsen, Kristian Nebelin Hvidt, Thomas A Schmidt

**Affiliations:** 1 Department of Emergency Medicine, Holbaek University Hospital, Denmark; 2 Department of Medicine, Holbaek University Hospital, Denmark

## Background

The Quick Diagnostic Unit (QDU) is an integrated part of the Emergency Department and patients admitted are expected to have a brief hospitalization. Understanding the discharge instructions is essential for patients' compliance. The objective of this study was to investigate comprehension of discharge instructions among elderly patients admitted to the QDU.

## Methods

A total of 102 adult patients discharged from the QDU answered a questionnaire covering self-assessed comprehension of discharge information, ability to recall discharge information and evaluation of the communication. Questions addressed diagnosis, diagnostic procedures, treatment, follow-up and return instructions. Answers from the questionnaire were compared with the discharge letter and the degree of concordance was evaluated. Patient awareness of own comprehension deficits was evaluated comparing self-assessed comprehension with the ability to recall a correct answer. The population was divided into two groups, an elderly group (age ≥ 65 years) and a younger group.

## Results

Forty patients were allocated to the elderly group and sixty-two to the younger group. The elderly group had more prior admissions compared to the younger group (P=0.027), whereas no difference was found for gender, education, other diseases or length of admission. Admission diagnosis in the total population was mainly anaemia, infectious or musculoskeletal disease. The range of self-assessed comprehension was 87.9 to 100% for the numerous conditions with no differences between the two groups. When compared with the discharge document the elderly group was less able to recall correct diagnostic procedures (91.94% vs. 71.79%, P=0.007) and medication instructions (77.97% vs. 54.29%, P=0.016). Furthermore, the elderly patients were less aware of their own comprehension deficits regarding diagnostic tests (P=0.006 / OR 0.95, 95% CI: 0.913-0.989, P=0.0115), preventive measures (P=0.015), medication instructions (P=0.028 / OR 0.95, 95% CI: 0.921 to 0.980, P=0.001) and when to seek emergency care (0.041).

## Conclusion

Elderly patients showed less ability to recall correct diagnostic procedures and medication instructions compared to younger patients. Furthermore they were less aware of their comprehension deficits. In this perspective, communicating with elderly patients requires special attention, this could involve "closed loops", repetition or follow up at a general practitioner.

